# Cloning and functional verification of the CmHSP17.9 gene from chrysanthemum

**DOI:** 10.1371/journal.pone.0301721

**Published:** 2024-05-08

**Authors:** Qin Ling, Shumei Zhang, Xin Li, Beibei Tang, Ai Chen, Tao Zeng, Qiqi Ma, Yijun Chen, Shaokang Tang, Yuanzhi Pan, Qinglin Liu, Yin Jia, Xue Yong, Beibei Jiang

**Affiliations:** 1 College of Landscape Architecture, Sichuan Agricultural University, Chengdu, China; 2 School of Landscape Architecture, Liaoning Agricultural College, Yingkou, China; University of Florida Institute of Food and Agricultural Sciences, UNITED STATES

## Abstract

Small molecular heat shock proteins (sHSPs) belong to the HSP family of molecular chaperones. Under high-temperature stress, they can prevent the aggregation of irreversible proteins and maintain the folding of denatured proteins to enhance heat resistance. In this study, the CmHSP17.9-1 and CmHSP17.9-2 genes, which were cloned from chrysanthemum (*Chrysanthemum×morifolium* ‘Jinba’) by homologous cloning, had a complete open reading frame of 480 bp each, encoding 159 amino acids. The protein subcellular localization analysis showed that CmHSP17.9-1 and CmHSP17.9-2 were located in the cytoplasm and mostly aggregated in granules, especially around the nucleus. Real-time quantitative PCR (qRT-PCR) analysis showed that the relative expression level of the CmHSP17.9-1 and CmHSP17.9-2 genes was highest in the terminal buds of the chrysanthemum, followed by the leaves. CmHSP17.9-1 and CmHSP17.9-2 overex-pression vectors were constructed and used to transform the chrysanthemum; overexpression of these genes led to the chrysanthemum phenotypes being less affected by high-temperature, and the antioxidant capacity was enhanced. The results showed that chrysanthemum with overex-pression of the CmHSP17.9-1 and CmHSP17.9-2 genes had stronger tolerance than the wild type chrysanthemum after high-temperature treatment or some degree of heat exercise, and overex-pression of the CmHSP17.9-1 gene led to stronger heat resistance than that of the CmHSP17.9-2 gene, providing an important theoretical basis for the subsequent molecular breeding and pro-duction applications of chrysanthemum.

## Introduction

Chrysanthemum (*Chrysanthemum×morifolium* Ramat) is a perennial flower of the genus chrysanthemum (*Chrysanthemum* L.) in the composite family (Asteraceae Bercht. J. Presl). The high ornamental value, medicinal value, edible value and economic value of chrysanthemum have allowed the chrysanthemum industry to endure [1, 2]. However, due to the trend of global warming in recent years, various physiological and biochemical changes in chrysanthemum under high-temperature stress, the molecular mechanism of heat tolerance and the improvement of heat tolerance have become popular research subjects and breeding objectives [3]. *Chrysanthemum×morifolium* ‘Jinba’ is a white chrysanthemum variety with strong adaptability [4]. The suitable temperature for its growth is 15℃-25℃. In the hot summer season, chrysanthemum plants readily undergo defoliation and prematurely aging [5].

High-temperature stress causes heat damage to plants, and plants respond accordingly to cope with the damage [6]. At this time, heat shock proteins (HSPs) are ex-pressed and accumulate in plant cells to prevent or reduce heat damage in plants [7]. The HSP family is a family of molecular chaperones, including HSP100, HSP90, HSP70, HSP60 and small molecular HSPs (sHSPs), which play an important role in resistance to heat stress in plants [8]. The small heat shock protein (sHSP) family includes some of the most diverse and complex proteins in the HSP family and plays an important role in plant stress resistance [9]. Under high-temperature stress, sHSP primarily performs molecular chaperone functions that prevent the clustering of irreversible proteins and maintain the folding of denatured proteins and is therefore also known as a house-keeping protein [10]. Some research results showed that the expression level of RcHSP17.8 in rose significantly increased after heat shock at 38℃ for 5 min, and it was transferred into *Arabidopsis thaliana*(L.) Heynh. and heat resistant transgenic plants were obtained [11]. Yang Meiling et al [12]. conducted gene cloning and genetic transformation of Arabidopsis, and found that the expression of MsHSP16.9 was strongly induced by high temperature. They also found that MsHSP16.9 may play a role in conjunction with AtHSP70 to maintain protein homeostasis and protect cells from damage. At present, there are no relevant research reports on small heat shock proteins in chrysanthemum; such a study would help to clarify the mechanism of high-temperature stress in chrysanthemum [13]. Research in this field will not only allow the development of corresponding measures to reduce the damage of high-temperature stress to chrysanthemum plants in a timely manner but also provide a theoretical basis for the introduction and cultivation, heat tolerance breeding and garden application of chrysanthemum, providing improved economic and ecological benefits [14, 15].

The purpose of this study was to isolate the CmHSP17.9–1 and CmHSP17.9–2 genes through homologous cloning and clarify their sequence characteristics, subcellular localization, physical and chemical properties, etc. The overexpression vector was constructed, chrysanthemum was transformed by Agrobacterium tumefaciens infection to obtain the CmHSP17.9–1 and CmHSP17.9–2 overexpression chrysanthemum, and the heat resistance function of CmHSP17.9–1 and CmHSP17.9–2 was identified.

## Materials and methods

### 2.1 Plant materials and cultivation conditions

The chrysanthemum in this study was planted in the tissue culture room of the School of Landscape Architecture, Sichuan Agricultural University. Routine maintenance and management were carried out according to the tissue culture conditions and specific needs. According to the conventional cultivation method, the seedlings were transplanted to plastic pots that were 7 cm×7 cm×13 cm in size when the plants were planted separately. Each pot contained one basic seedling under routine management.

### 2.2 Gene cloning and sequence analysis

In the experiment, Trizol reagent was used to extract total RNA from the leaves of chrysanthemum plants, 1% agarose gel was used to detect RNA degradation and pollution, and the reverse transcriptase of the HiScript III first strand cDNA synthesis kit was used to convert it into cDNA. And then, homologous primers (see S1 Table) were used to perform PCR procedure for the reverse cDNA through a thermal cycle instrument (T100, BIO-RAD). Then sequenced.

Tobacco(*Nicotiana tabacum* L.) small heat shock protein sequence is from the NCBI public database of the website of the National Biotechnology Information Center of the United States (http://www.ncbi.nlm.nih.gov/). The cDNA sequence was translated with DNAman5.0 software and the protein sequence was analyzed for similarity. The conserved domain of protein was analyzed by NCBI (https://www.ncbi.nlm.nih.gov/). The contents of various amino acids in chrysanthemum CmHSP17.9–1 and CmHSP17.9–2 genes were counted by ProtParam program of online software Expasy (https://web.expasy.org/protparam/), and their theoretical molecular weight and isoelectric point were predicted. Adopt ProtScale program (http://web.expasy.org/protscale/) to conduct hydrophobicity analysis of CmHSP17.9–1 and CmHSP17.9–2. Application of online tool PSIPRED for protein secondary structure prediction of CmHSP17.9–1 and CmHSP17.9–2 (https://www.novopro.cn/tools/secondary-structure-prediction.html). Adopt online tool WoLF PSORT—Protein Subcellular Localization Prediction (https://wolfpsort.hgc.jp/) and ProtComp v.9.0 database (http://www.softberry.com/) to predict the subcellular localization characteristics of proteins. Online tool SignalP is used for protein signal peptide prediction (http://www.detaibio.com/tools/signal-peptide.html). Transmembrane information prediction: (www.detaibo.com/tools/transmembrane.html). Retrieve the sequence highly similar to CmHSP17.9–1 and CmHSP17.9–2 protein sequences in NCBI database (https://blast.ncbi.nlm.nih.gov/Blast.cgi), and then using MEDA software, through Neighbor-Joining (NJ) to build phylogenetic tree, and the internal branch Bootstrap detection value is set to 1000 times.

### 2.3 Gene subcellular location analysis

For subcellular localization analysis, the tobacco transient expression vectors for CmHSP17.9 were constructed, and the subcellular localization observation experiment with transient transformation was carried out in tobacco leaves and tobacco protoplasts. The experiment was commissioned by Shanghai Zhishuo Biotechnology Co., Ltd. and Wuhan Boyuan Biotechnology Co., Ltd.

Instantaneous transformation experiment of tobacco leaves: the plasmid liquid nitrogen freeze-thaw method was used to transform Agrobacterium GV3101, and the strain was saved after the colony PCR identification was correct. Agrobacterium tumefaciens cultured overnight were centrifugally suspended, and the suspension composition (10 mM MgCl_2_, 10 mM MES, 100 *μ*M AS), and inject 4 5 leaves of Bunji tobacco after standing for 3 h at room temperature. The injected tobacco was observed by laser confocal microscope 3 days later.

Instantaneous transformation experiment of tobacco protoplast: the constructed vector plasmid was transferred into Agrobacterium tumefaciens GV3101 and cultured at 30℃ for 2 days. Scrape the Agrobacterium tumefaciens from the solid culture dish with the inoculum ring, and then put it into the 10mL YEB liquid medium, and cultivate it for 1h, centrifugation for 4 min with 1776 g, and then remove the supernatant. Use 10 mM MgCl_2_ (containing 120 *μ*M AS) to resuspend the suspension, adjust OD600 to about 0.6. Select tobacco plants with good growth status, inject them from the lower epidermis of tobacco leaves with a 1 mL syringe without the gun head, and mark them. The injected tobacco plants were cultured in low light for 2 days, and then observed. Take the labeled tobacco leaves injected with Agrobacterium, make them into slides, observe them under the laser confocal microscope, and take photos. Cut the fluorescent tobacco leaves into about 1 mm segments, add 5–10 mL of enzymolysis solution, and soak all the tissues. Enzymatic hydrolysis at 24℃ for 1 h. After filtering with filter screen, centrifuge at 9.99 g for 3 min, and remove the supernatant. Wash twice with 10 mL of precooled W5 solution, centrifuge at 9.99 g for 3 min, and centrifuge at 4–25℃. Add a certain amount of MMG solution suspension as required. Observe with laser confocal microscope.

### 2.4 Genetic transformation of genes in the chrysanthemum

The plant expression vector pCAMBIA2301-HSP17.9 was constructed, and the plant expression vector was transferred to Agrobacterium tumefaciens. The plasmid was extracted, digested and sequenced, and the chrysanthemum plant was transformed by Agrobacterium tumefaciens infected method. PCAMBIA2301-HSP17.9 vector was used for genetic transformation to study the function of related genes. The overexpression vector pCAMBIA2301-HSP17.9 was transformed into Agrobacterium strain GV3101 by freeze-thaw method, and the positive clones were screened. Agrobacterium tumefaciens-mediated leaf-disk infection was used to transform chrysanthemum. Extract the DNA of transgenic chrysanthemum positive strains as a template, design primers (see S2 Table) according to the gene, carry out PCR amplification, select the positive strains that can amplify fragments, extract RNA and reverse transcription, and obtain the cDNA for real-time fluorescence quantitative detection. The transgenic strains with relatively high expression level were selected for a large number of propagation, and then the seedlings were acclimatized, domesticated and transplanted after rooting.

### 2.5 Analysis of gene expression characteristics

Sampling for tissue-specific expression analysis: The roots, stems, leaves and terminal buds of the chrysanthemum plants were selected and stored at -80℃ after quick freezing with liquid nitrogen.

High-temperature treatment and sampling: chrysanthemum tissue culture seedlings with 6–8 leaves were transplanted into disposable plastic cups for high-temperature treatment, and the cultivation medium was a 1:1 mixture of vermiculite and peat soil. High-temperature stress treatment was carried out after 8–10 leaves of the plant were unfurled. Chrysanthemum plants with the same growth trend were thoroughly watered and then placed in a light incubator (temperature of 42°C, relative humidity of 30%) for high-temperature stress. The test treatment time was 6 hours. The sampling time points were 0 h, 1 h, 3 h and 6 h after high-temperature treatment, and the sampling position was the fourth to fifth leaf from the top leaf of the plant. Each treatment was repeated with three individual plants, and then the samples were frozen in liquid nitrogen and stored at -80°C [16].

The expression of the CmHSP17.9–1 and CmHSP17.9–2 genes was measured and analyzed by qRT-PCR and relative quantitative analysis. The cDNA of chrysanthemum leaves was used as a template, B-actin was used as an internal reference gene, and the primers used are shown in S3 Table.

### 2.6 Acclimatizing cultivation, high-temperature treatment and physiological index determination of transgenic plants

Transgenic strains with high expression were selected for subculture. Transgenic chrysanthemum tissue culture seedlings with good root development were selected, the cover was opened, and the seedlings were acclimatized in the culture room for 5 days, transplanted to flowerpots, cultivated in a light incubator for 7 days, and finally transplanted in a greenhouse for normal management. At the same time, well-growing nontransgenic chrysanthemum tissue culture seedlings were selected as the control, and the acclimatizing cultivation method was the same as above.

The treatment method of high-temperature stress is the same as 2.5. After 0 h, 3 h and 6 h of high-temperature stress [16], the physiological indexes such as electrolyte permeability, chlorophyll content, free proline content, soluble sugar content, MDA content, antioxidant enzyme activity and reactive oxygen accumulation were measured in functional leaves of the same size. Data analysis shall be conducted after the measurement of indicators.

The accumulation of hydrogen peroxide (H_2_O_2_) and superoxide anion (${\text{O}}_{2}^{-}$) in leaves was determined by histochemical staining [17, 18]. Chlorophyll content was determined by soaking in ethanol-acetone mixture [19]. The relative permeability of the cell membrane was measured using the conductivity method. The content of MDA and soluble sugar was determined by thio Barbituric acid colorimetry (TBA) [20]. The content of free proline was determined by acidic ninhydrin colorimetry [21]. The activity of SOD enzyme was determined by the photochemical reduction method of NBT [22]. The activity of POD enzyme activity was determined by guaiacol method [23]. The activity of CAT and APX enzyme activity was determined by ultraviolet spectrophotometry [24].

### 2.7 Data analysis

The test data were analyzed by one-way ANOVA using SPSS23.0 statistical software, and the multiple comparisons of the means were conducted by one-way ANOVA test (p = 0.05), and plotted using Microsoft Excel 2013 software.

## Results

### 3.1 Gene cloning and sequence analysis

**Fig 1:**
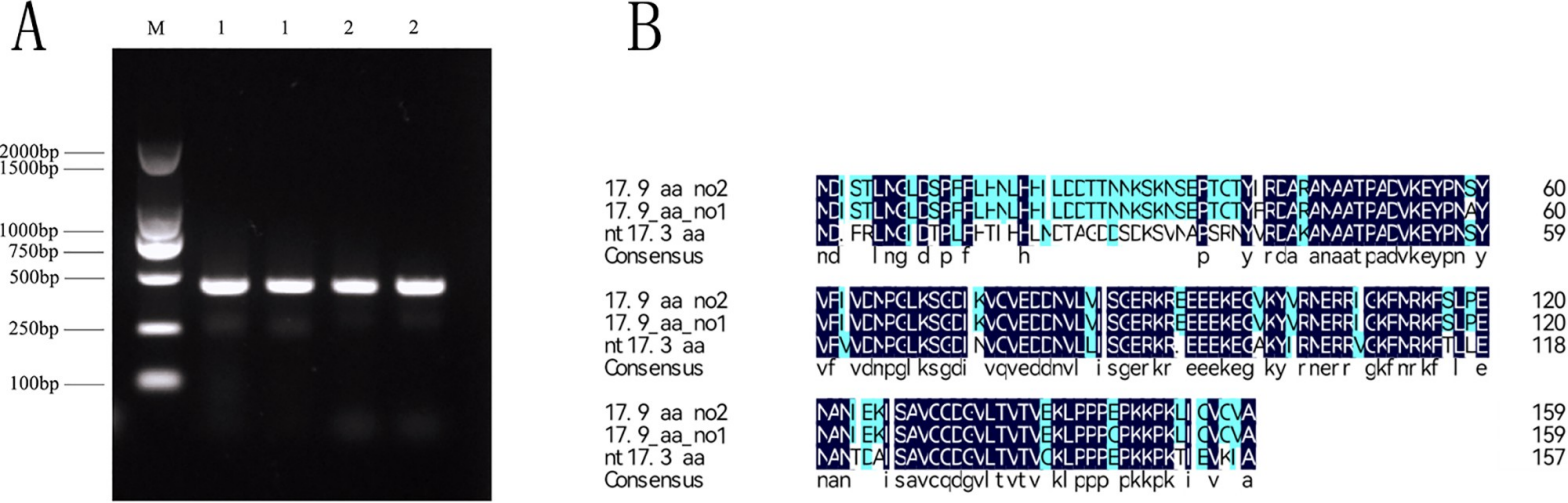
Gene cloning and sequence analysis of HSP17.9. (A)RT-PCR product electrophoresis map of HSP17.9 gene(M: DNA Labber marker; 1: CmHSP17.9–1 PCR product; 2: CmHSP17.9–2 PCR product); (B) Comparison of amino acid sequences of CmHSP17.9–1, CmHSP17.9–2 and tobacco HSP17.3 genes.

Chrysanthemum cDNA was amplified as a template with specific primers, and then the PCR product was detected with 1% gel electrophoresis; two specific bands were obtained Fig 1A. The specific bands were recovered and connected to the T vector for sequencing.

After sequencing and verification, the CmHSP17.9–1 and CmHSP17.9–2 CDS sequences of chrysanthemum were obtained. The total length of the ORF region sequence of the target gene was 480 bp, encoding 159 amino acids, and the molecular weight was about 17.9 kDa. According to the molecular weight, they were named as CmHSP17.9–1 and CmHSP17.9–2 genes, and they were registered in NCBI with the login numbers of ON692933 and ON692934 respectively.

The results of amino acid sequence alignment of the two cloned genes showed that there were two different amino acids in the two cloned sequences, which might be that the two clones belonged to alleles. The results of amino acid sequence comparison with tobacco HSP17.3 gene showed that compared with tobacco HSP17.3 amino acid sequence, the N-terminal sequence was significantly different and the C-terminal sequence was relatively high homology(Fig 1B).

**Fig 2:**
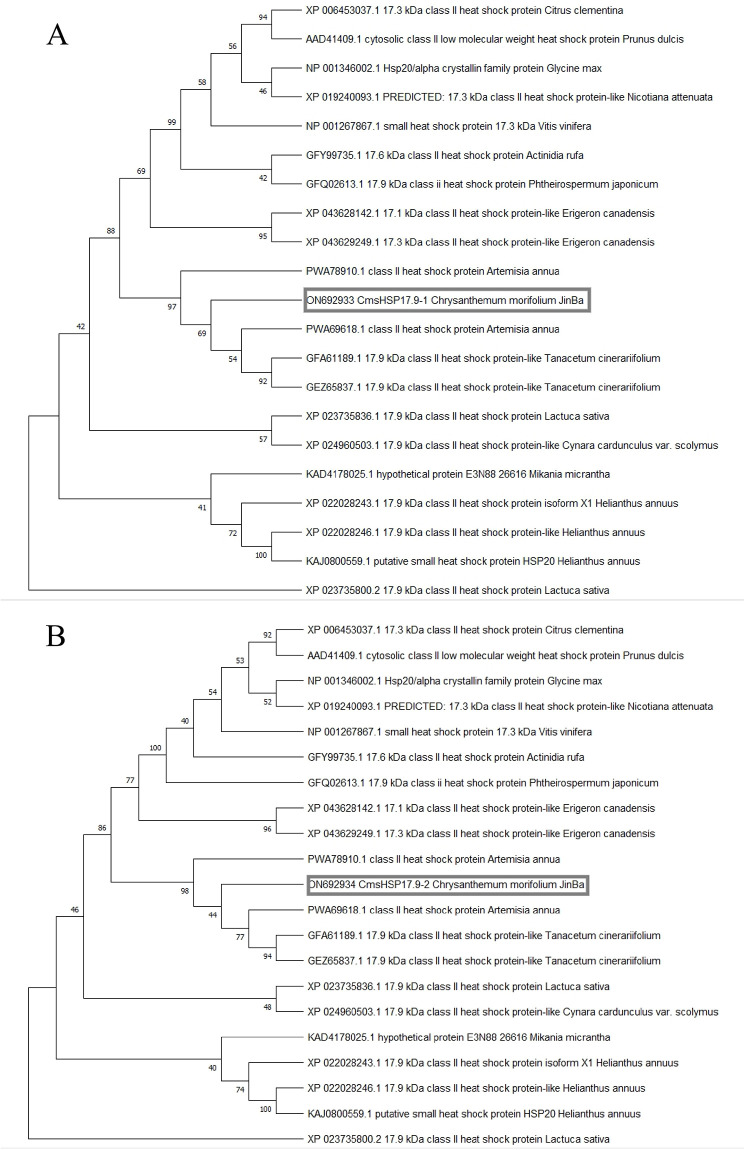
Cluster analysis of amino acid sequences encoded by CmHSP17.9. (A) CmHSP17.9–1 gene phylogenetic tree; (B) CmHSP17.9–2 gene phylogenetic tree.

MEGA software was used to carry out multiple sequence alignment analysis of the CmHSP17.9–1 and CmHSP17.9–2 genes and HSP17.9 amino acid sequences in other species and to construct a phylogenetic tree (Fig 2). The results showed that the 10.1–18.1 kDa small molecular heat shock protein family members of Artemisia annua and Tanacetum cinerariifolium in the composite family and CmHSP17.9 were located in the same branch of the phylogenetic tree. This branch harboured the cytoplasmic class II (CII) small molecular heat shock proteins. Among them, CmHSP17.9–1 and CmHSP17.9–2 exhibited higher homology with HSP18.1, a CII member of Artemisia annua (Fig 2). CmHSP17.9–1 and CmHSP17.9–2 are CII small molecular heat shock proteins. CII-type small molecular heat shock proteins are located in the nucleus or cytoplasm and often participate in multiple stress responses, such as responses to salt, high-temperature, low-temperature and oxidative stress, suggesting that the CmHSP17.9–1 and CmHSP17.9–2 genes of chrysanthemum may participate in regulating the response to abiotic stress, such as high-temperature, in chrysanthemum and play a very important role in the process of plant resistance to abiotic stress [25].

### 3.2 Analysis of gene bioinformatics

Through online tools predict and analyze 159 amino acids encoded by CmHSP17.9–1 and CmHSP17.9–2 genes.

The results show that the theoretical isoelectric point of CmHSP17.9–1 gene is 6.32, the molecular formula is C_797_H_1284_N_218_O_242_S_7_, and the total number of atoms is 2548. The total number of negatively charged residues (Asp+Glu) is 24, and the total number of positively charged residues (Arg+Lys) is 23. The instability index of protein is 45.81, which is considered as unstable protein. The average hydrophilicity coefficient is -0.519, which is hydrophilic protein.

**Fig 3:**
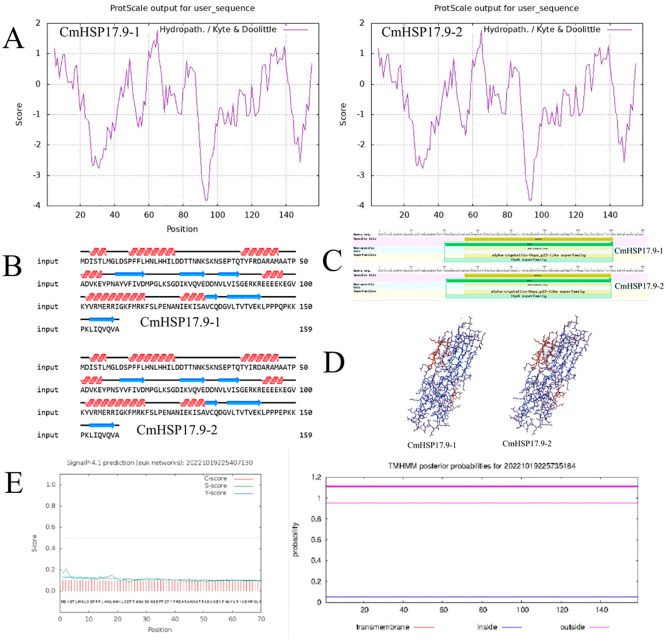
Bioinformatics analysis of CmHSP17.9. (A)Hydrophilic analysis; (B) Prediction of secondary structure; (C)Conserved domains analysis; (D)tertiary structure prediction; (E)Prediction of the signal peptide and transmembrane structure.

The theoretical isoelectric point of CmHSP17.9–2 gene is 5.96, the molecular formula is C_794_H_1285_N_217_O_244_S_7_, and the total number of atoms is 2547. The total number of negatively charged residues (Asp+Glu) is 25, and the total number of positively charged residues (Arg+Lys) is 23. The instability index of protein is 45.69, which is considered as unstable protein. The average hydrophilicity coefficient is -0.525, which is hydrophilic protein (Fig 3A).

The online tool PSIPRED was used to predict the secondary structure of the CmHSP17.9–1 and CmHSP17.9–2 proteins of chrysanthemum, and the results showed that the CmHSP17.9–1 and CmHSP17.9–2 secondary structures exhibited the following composition: a-alpha helix, 34.18%; random coil, 43.67%; and extended strand, 22.15%. This shows that the helical structure and random coil were the main components of the secondary structure of the protein. The specific combination of different types of conformations is shown in the figure below (Fig 3B).

According to the conserved domain analysis of proteins in the NCBI database (Fig 3C and 3D), the positions 52 141 amino acid region of CmHSP17.9–1 and CmHSP17.9–2 was the typical ACD (alpha crystal domain) of the HSP20 subfamily small molecular heat shock protein (pfam00011). The positions 40 141 amino acid region was similar to the COG0071 domain of the molecular chaperone protein IbpA. IbpA is a member of the small molecular heat shock protein family [26]. IbpA and IbpB work together to protect the substrate protein in the heat shock response by eliminating agglutination, producing refolding and restoring activity [27].

### 3.3 Gene subcellular localization analysis and signal peptide prediction

The signal peptide prediction analysis of the CmHSP17.9–1 and CmHSP17.9–2 proteins was carried out using SignalP online software, and the results showed that they did not contain signal peptides, had no transmembrane structure and were not extracellular secreted proteins or membrane proteins(Fig 3E).

**Fig 4:**
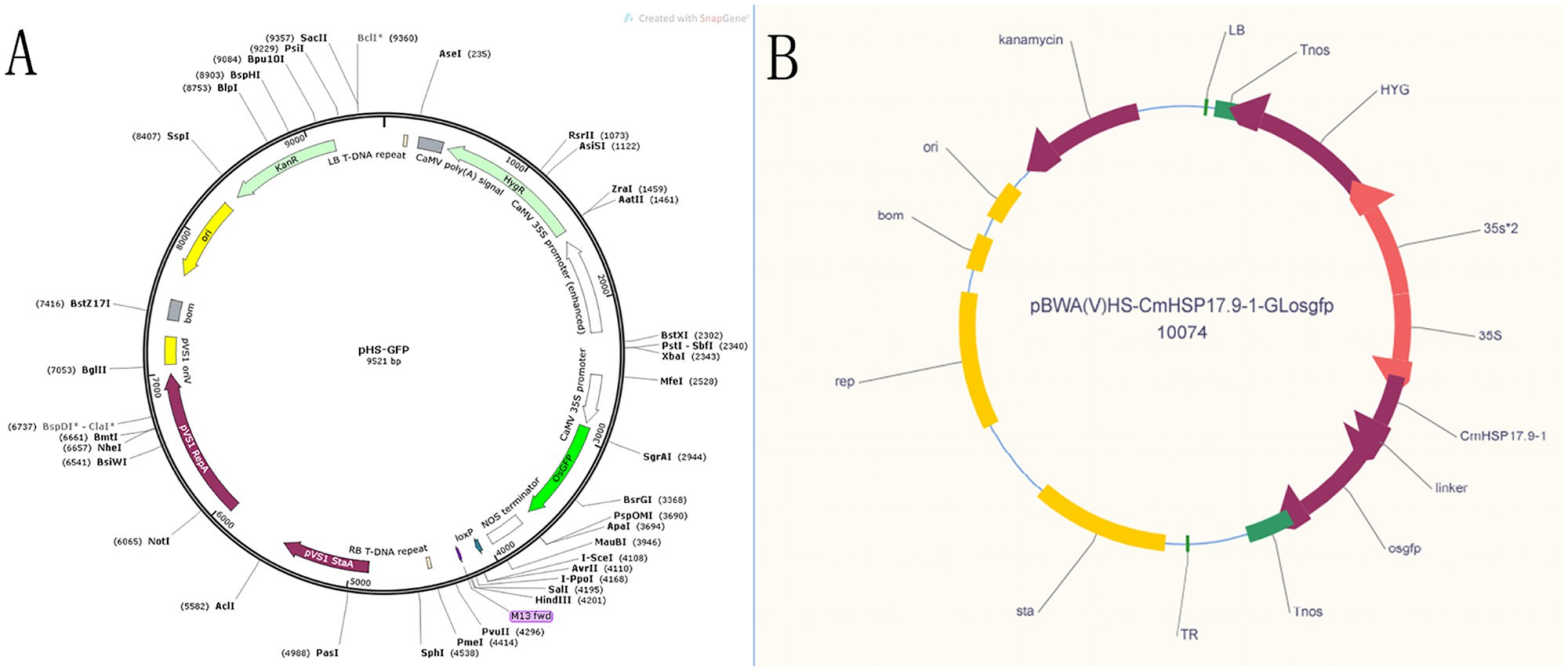
(A) CmHSP17.9 HS-GFP vector map of subcellular localization in tobacco leaf; (B) CmHSP17.9 HS-GFP vector map of subcellular localization in tobacco protoplast.

To further understand the subcellular localization of the CmHSP17.9–1 and CmHSP17.9–2 proteins, HS-CmHSP17.9-GFP fusion protein expression vectors were constructed (see S4 Table and Fig 4 for vector map), and subcellular localization experiments via tobacco leaf transient transformation and tobacco protoplast transient transformation were carried out.

**Fig 5:**
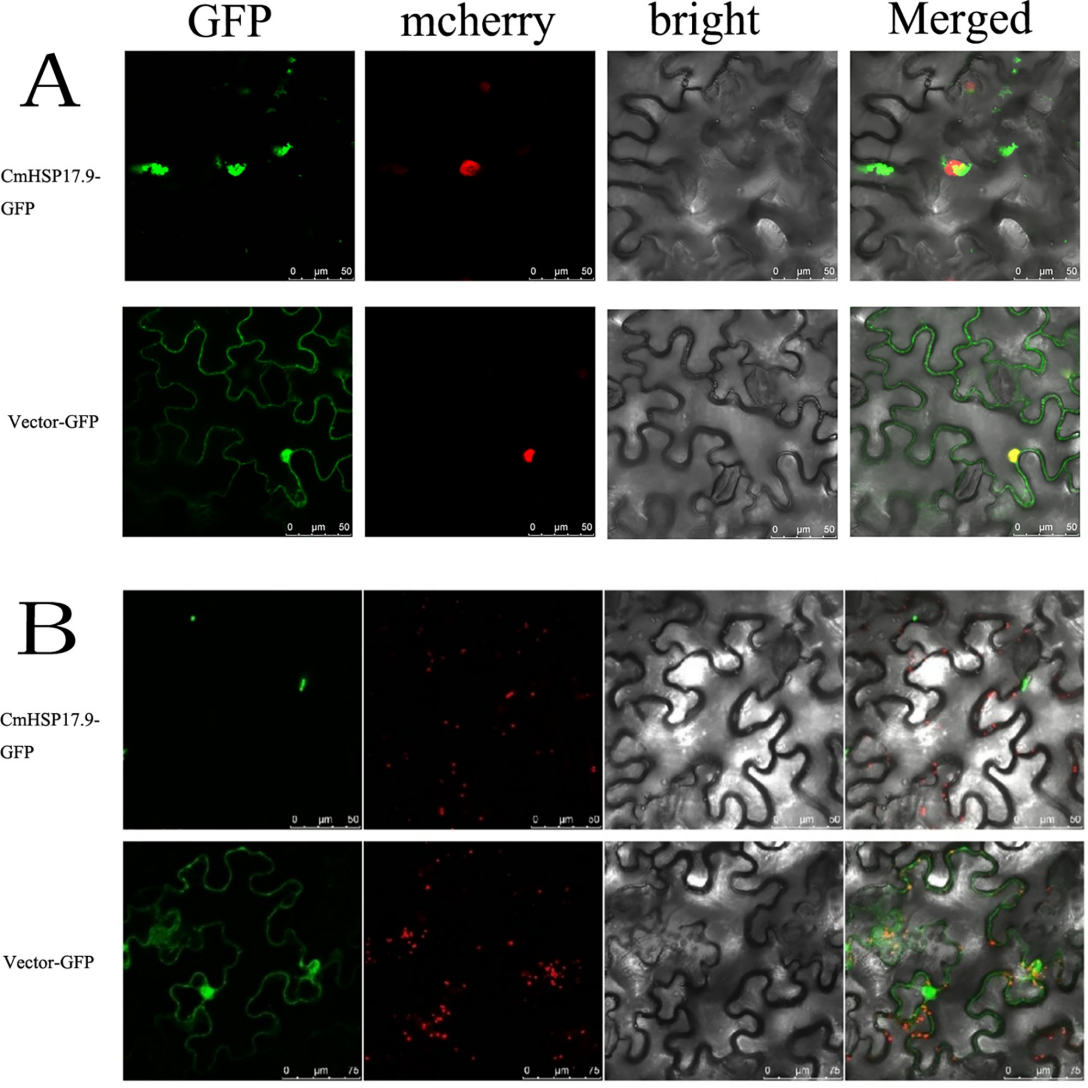
Subcellular localization of CmHSP17.9. (A) Transient expression of genes in tobacco leaf cells labeled with nuclear markers; (B)Transient expression of genes in tobacco leaf cells labeled with peroxisome markers.

First, the observation results in tobacco leaves (Fig 5A) showed that the GFP gene in the tobacco leaf cells encoded on the empty vector 35S-GFP was constitutively expressed, and green fluorescence was distributed in both the nucleus and cytoplasm. The fluorescence signal in the nucleus was stronger than that in the cytoplasm, and the fluorescence distribution was relatively uniform in both the nucleus and the cytoplasm. However, the green fluorescence of the CmHSP17.9-GFP fusion protein did not overlap with the nuclear marker, indicating that it was not distributed in the nucleus, only in the cytoplasm. The fluorescence was not evenly distributed, and there was aggregation in the cytoplasm. It is speculated that this aggregation may have been the peroxisome, Golgi apparatus, or mitochondria. Then, observation with the Perox marker was carried out (Fig 5B). The results showed that the green fluorescence of the CmHSP17.9-GFP fusion protein was significantly weaker than that of organelles, its shape was irregular, and the signal did not overlap with that of the Perox marker. Therefore, the CmHSP17.9 protein was not located in the peroxisome and was less likely to be present in other organelles. According to the results of observations with two markers, the CmHSP17.9-GFP fusion protein was located in the cytoplasm and was mostly aggregated in granules, especially around the nucleus.

**Fig 6:**
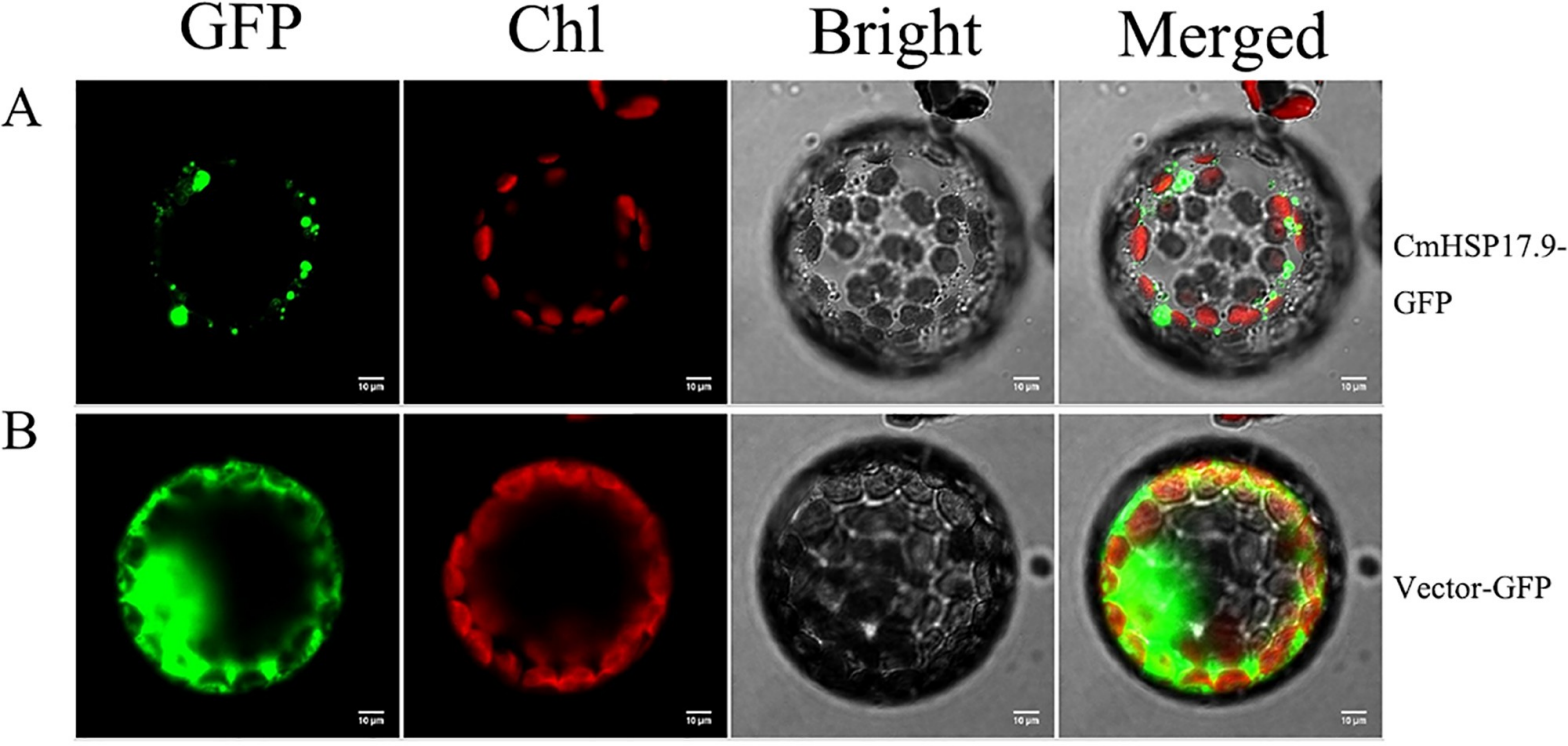
Transient expression of genes in tobacco protoplast cells.

To verify the reliability of the experimental results, the constructed subcellular localization recombinant vector CmHSP17.9-GFP and the 35S-GFP empty vector were transformed into tobacco protoplasts, and the results were observed after 18 hours (Fig 6). The localization results were the same as those in tobacco leaf cells. The target gene was expressed in tobacco prairie protoplasts in granular form. This result was consistent with the previous localization study of OsHSP20 and NbHSP20 in tobacco, which showed that the granular structure formed by OsHSP20 and NbHSP20 in Nicotiana benthamiana could move and the original granular fluorescent aggregate could disappear with the change.

### 3.4 Identification of CmHSP17.9–1 and CmHSP17.9–2 transgenic chrysanthemum

#### 3.4.1 Molecular identification of transgenic chrysanthemum

**Fig 7:**
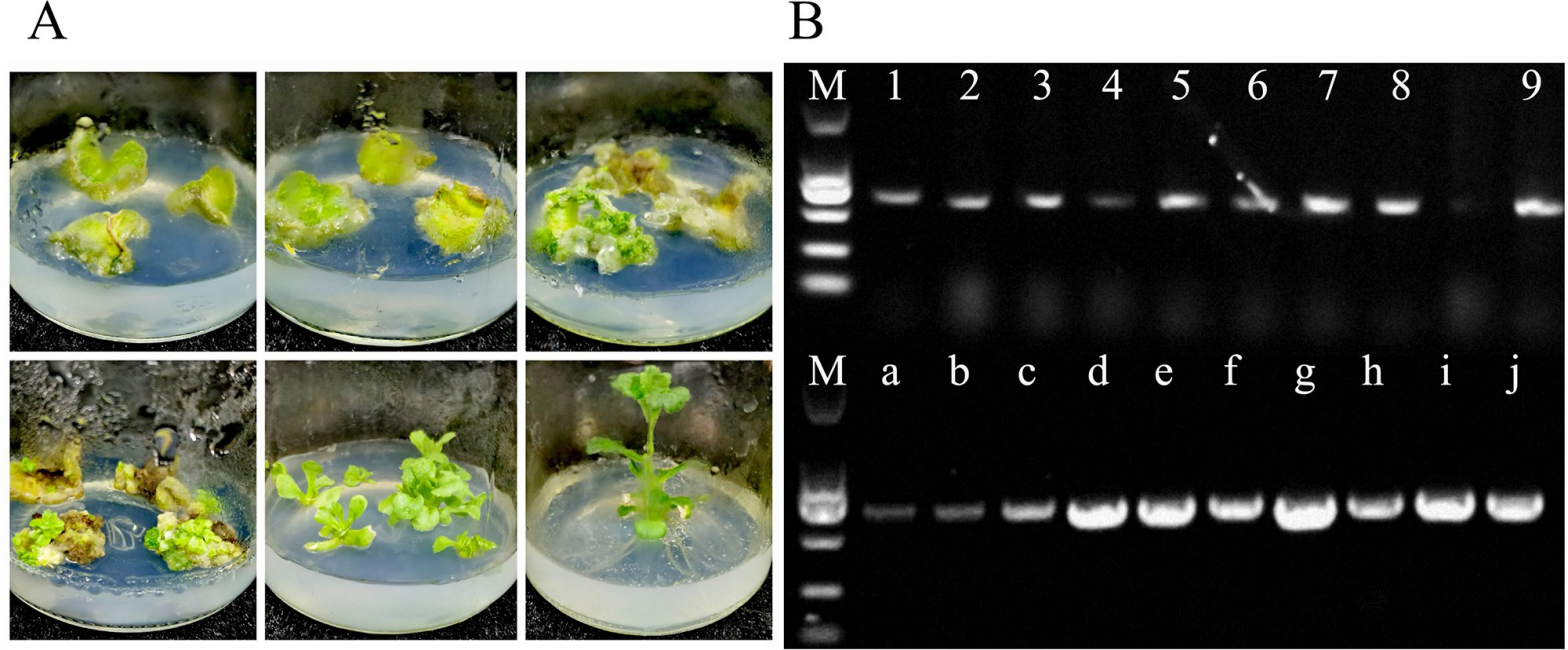
Genetic transformation of CmHSP17.9 gene and identification of positive seedlings. (A) Genetic transformation of CmHSP17.9–1 and CmHSP17.9–2 in the chrysanthemum; (B) Electrophoresis patterns of CmHSP17.9–1 and CmHSP17.9–2 positive strains were identified (M: DNA Labber marker; 1–9: Identification of CmHSP17.9–1 Gene Positive Strains; a-j: Identification of CmHSP17.9–2 Gene Positive Strains).

**Fig 8:**
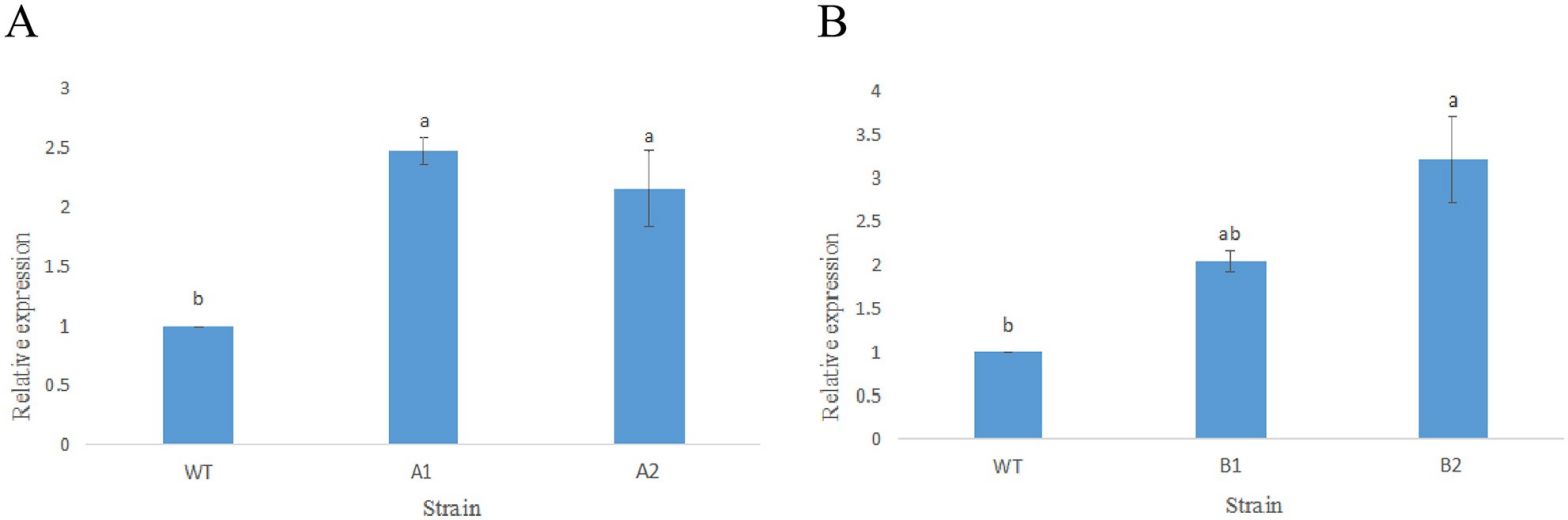
Gene expression levels in transgenic strains. (A) CmHSP17.9–1; (B) CmHSP17.9–2.

The transgenic overexpression strains were obtained by transformation of CmHSP17.9–1 and CmHSP17.9–2 in chrysanthemum through the leaf-disk infection method (Fig 7A). Primers were designed according to the desired gene fragments (S2 Table), and DNA was extracted by the CTAB method. 1× T3 Super PCR Mix was used to identify transgenic seedlings by PCR. As shown in Fig 8B, the four transgenic strains had specific bands, and the positive control with plasmid pCAMBIA2301-HSP17.9 as a template had targeted bands.

The expression of CmHSP17.9–1 and CmHSP17.9–2 were analyzed by quantitative real-time PCR. The CmHSP17.9–1 transgenic strains A1 and A2, and the CmHSP17.9–2 transgenic strains B1 and B2 were selected for subsequent experiments due to high expression. As shown in Fig 8, there are significant differences in the expression of CmHSP17.9–1 and CmHSP17.9–2 genes between the overexpression strains and the wild-type strains, and the relative expression of the four positive strains is significantly higher than that of the control plants.

#### 3.4.2 GUS staining identification of transgenic chrysanthemum

**Fig 9:**
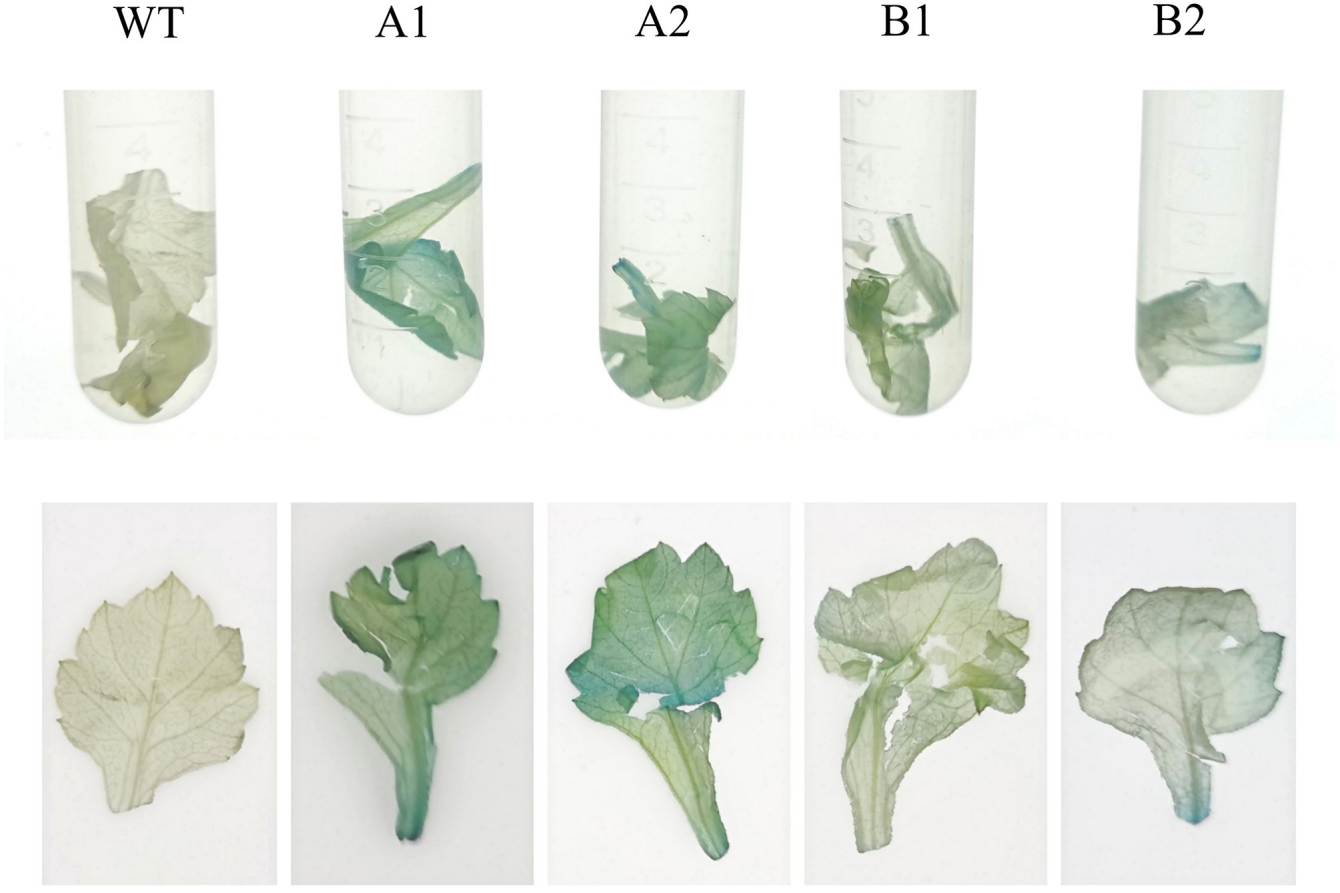
GUS staining of wild-type strains and transgenic strains. (This figure © 2024 by Qin Ling et al. is an open access article distributed under the terms of the Creative Commons Attribution License, which permits unrestricted use, distribution, and reproduction in any medium, provided the original author and source are credited).

GUS staining was carried out on plant samples [28]. Because most plant cells do not have endogenous GUS activity, the GUS gene is widely used as a reporter gene in transgenic plants [29]. These results were consistent with the molecular identification study, and the four transgenic lines showed obvious blue colour compared with the control(Fig 9).

### 3.5 Analysis of gene expression characteristics

#### 3.5.1 Expression characteristics of CmHSP17.9–1 and CmHSP17.9–2 in different tissues and organs

**Fig 10:**
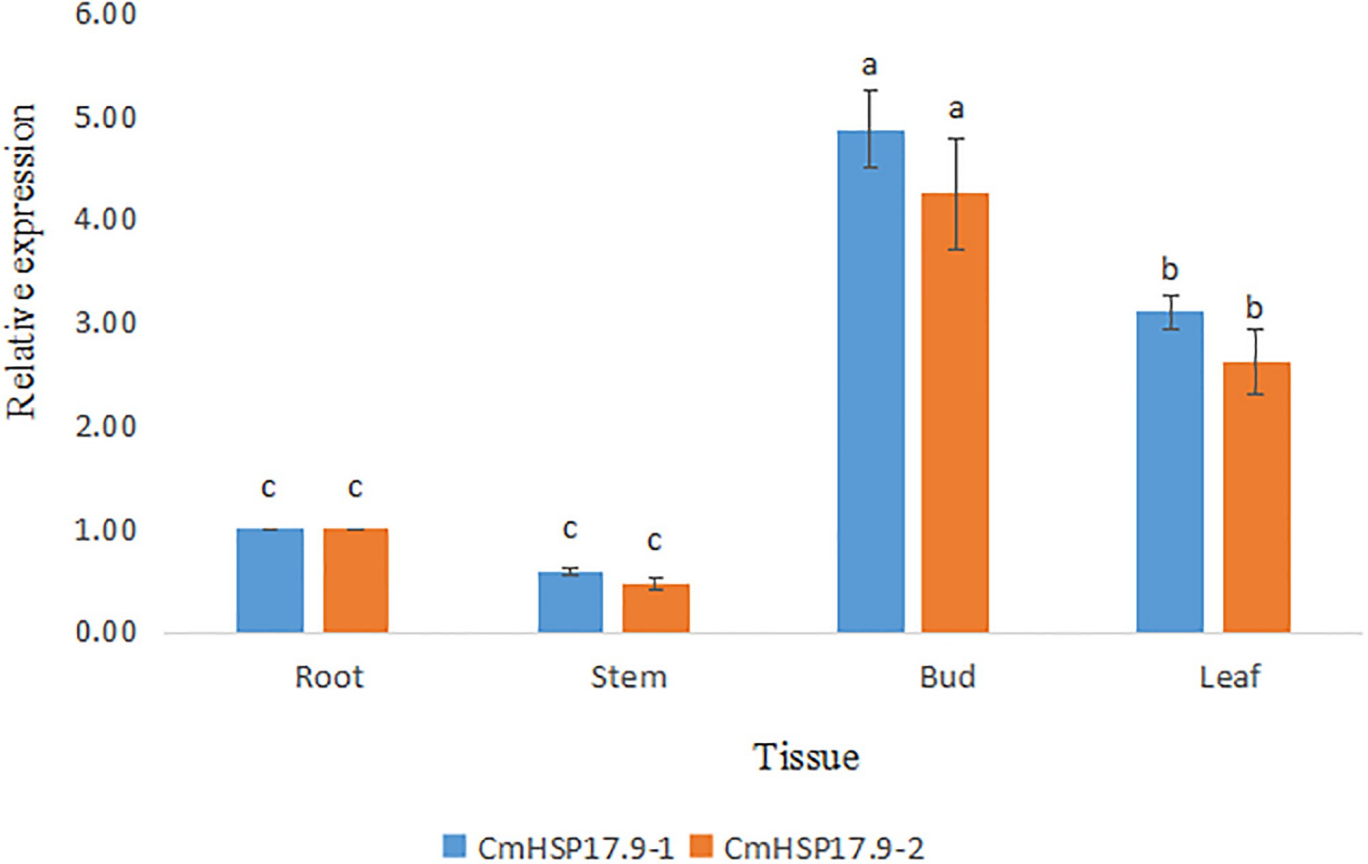
Expression of CmHSP17.9–1 and CmHSP17.9–2 in different tissues of chrysanthemum.

As shown in Fig 10, the expression levels of CmHSP17.9–1 and CmHSP17.9–2 varied greatly among different organs. The expression of CmHSP17.9–1 and CmHSP17.9–2 in the stem was the lowest, 0.58 times and 0.47 times that in the root, respectively. The relative expression in the terminal bud was the highest, 4.87 times and 4.25 times that in the root, respectively, followed by the leaves, where it was 3.10 times and 2.62 times that in the stem, respectively.

#### 3.5.2 Expression analysis of CmHSP17.9–1 and CmHSP17.9–2 under high-temperature stress

**Fig 11:**
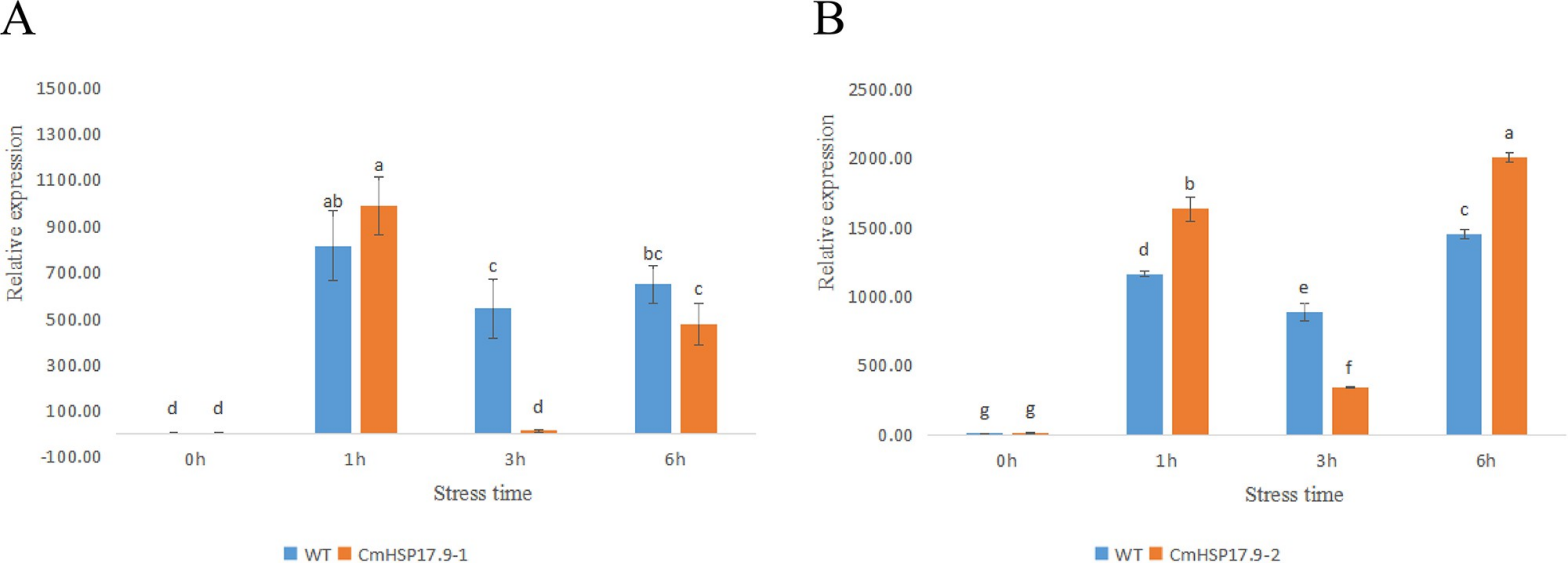
Changes in CmHSP17.9 expression levels in chrysanthemum under high-temperature stress. (A) CmHSP17.9–1; (B) CmHSP17.9–2.

To explore the expression patterns of CmHSP17.9–1 and CmHSP17.9–2 in chrysanthemum under heat stress, qRT-QCR analysis was carried out. The results showed that the expression of CmHSP17.9–1 was continuously induced under heat stress and peaked at 1 h, when it was 990.36 times that in the nonstressed control group. Then, the induced expression level showed a downwards trend, but it was still higher than the control level until 6 h. At 6 h, the expression level in the heat stress treatment group was 478.02 times that in the nonstressed control group (Fig 11A).

Similarly, under heat stress, CmHSP17.9–2 expression was continuously induced, with a significant upwards trend at 1 h, when the expression level was 1177.27 times that in the nonstressed control group. Then, the induction level showed a downwards trend but continued to rise at 6 h, reaching 1807.83 times the control level (Fig 11B).

The expression pattern analysis showed that heat stress could rapidly induce the expression of CmHSP17.9–1 and CmHSP17.9–2, and the expression of the two genes could rapidly respond to high-temperature stress within 1 h. However, high-temperature only induced mRNA accumulation in a short period of time, and express did not continue indefinitely with the extension of stress duration, so the expression of the two genes showed a trend of rising first and then falling. After high-temperature induction for a certain duration, the induction of gene expression was inhibited, and mRNA was degraded.

### 3.6 Analysis of phenotypic and physiological changes in chrysanthemum plants under high-temperature stress

#### 3.6.1 Analysis of phenotypic changes in chrysanthemum under high-temperature stress

**Fig 12:**
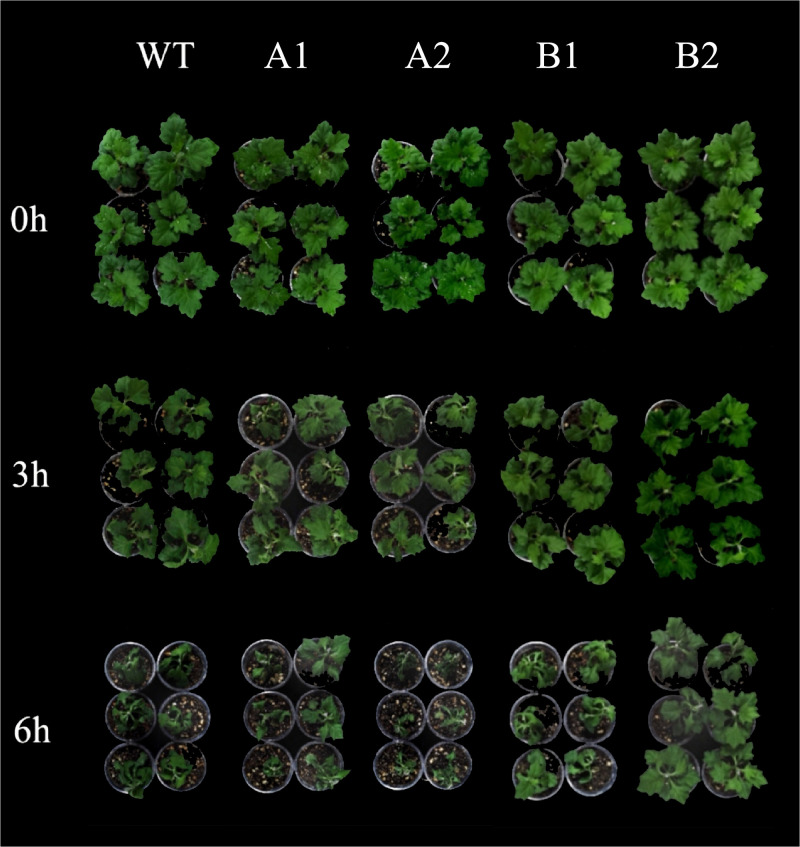
Phenotypic changes of wild-type strains and transgenic strains under high-temperature treatment. (A1 and A2 are the CmHSP17.9–1 transgenic strains; B1 and B2 are the CmHSP17.9–2 transgenic strains).

The obtained overexpression strains of the CmHSP17.9–1 and CmHSP17.9–2 genes were subjected to high-temperature stress. The results showed that the apparent morphology of different strains of chrysanthemum changed significantly under the same environmental stress. After 3 hours of high-temperature treatment, the leaves of the wild-type strains all wilted and drooped, while the leaves of the four overexpression strains showed no obvious drooping. After 6 hours, the leaves of wild-type chrysanthemum became thinner, wilted and drooped more severely. For the transgenic strains, the lower leaves of the plants were wilted, while the upper leaves remained tall and straight, and the leaves were basically fully extended, except for the A2 strain. However, due to the short stress duration, the chlorosis of the leaves of each plant strain was not obvious. The above results showed that the transgenic strains were less affected by high temperature. Overexpression of the CmHSP17.9–1 and CmHSP17.9–2 genes in chrysanthemum improved the high-temperature tolerance of chrysanthemum (Fig 12).

#### 3.6.2 Changes in chlorophyll content in chrysanthemum leaves under high-temperature stress

**Fig 13:**
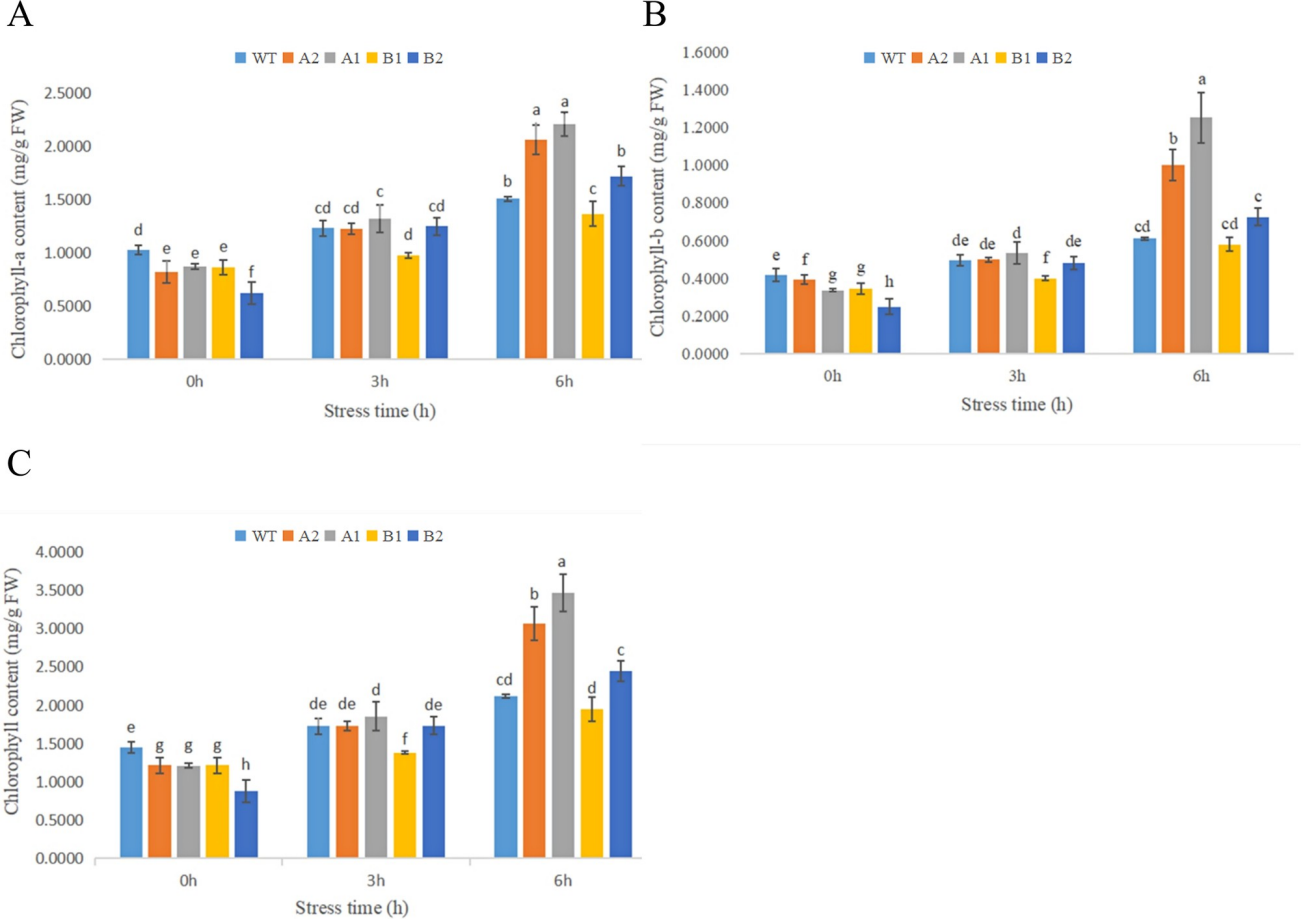
Changes in the Chlorophyll, Chlorophyll A and Chlorophyll B content of chrysanthemum under high-temperature treatment. (A) Chlorophyll; (B)Chlorophyll A; (C) Chlorophyll B.

As shown in Fig 13, the chlorophyll content showed an upwards trend after high-temperature stress. Compared with the control, the two transgenic strains of CmHSP17.9–1 showed no significant difference in the increase in chlorophyll content after 3 h of stress but showed a significant increase after 6 h of stress. Compared with the control, the chlorophyll content of the CmHSP17.9–2 transgenic strain B2 increased significantly after 6 h of stress, while the chlorophyll content of strain B1 was not significantly different. The levels of chlorophyll A and chlorophyll B increased after high-temperature stress. The difference between the transgenic strains and the control was similar to the chlorophyll content. The ratio of chlorophyll A content to chlorophyll B content was approximately 1.5–2.5. It can be inferred from the above results that the chlorophyll content of chrysanthemum seedlings did not decrease due to the short stress duration, and the plants may increase the chlorophyll content in response to heat shock. This is consistent with the phenotypic observations.

The chlorophyll content showed an upwards trend. It was speculated that the chlorophyll content increased temporarily due to the limited duration of stress, but the chlorophyll content of the four transgenic strains increased significantly compared with the control, indicating that under high-temperature stress, the chlorophyll content of transgenic chrysanthemum was less strongly affected and the ability to maintain chlorophyll was stronger than that of wild-type chrysanthemum.

#### 3.6.3 Changes in the MDA content and relative cell membrane permeability of chrysanthemum under high-temperature stress

**Fig 14:**
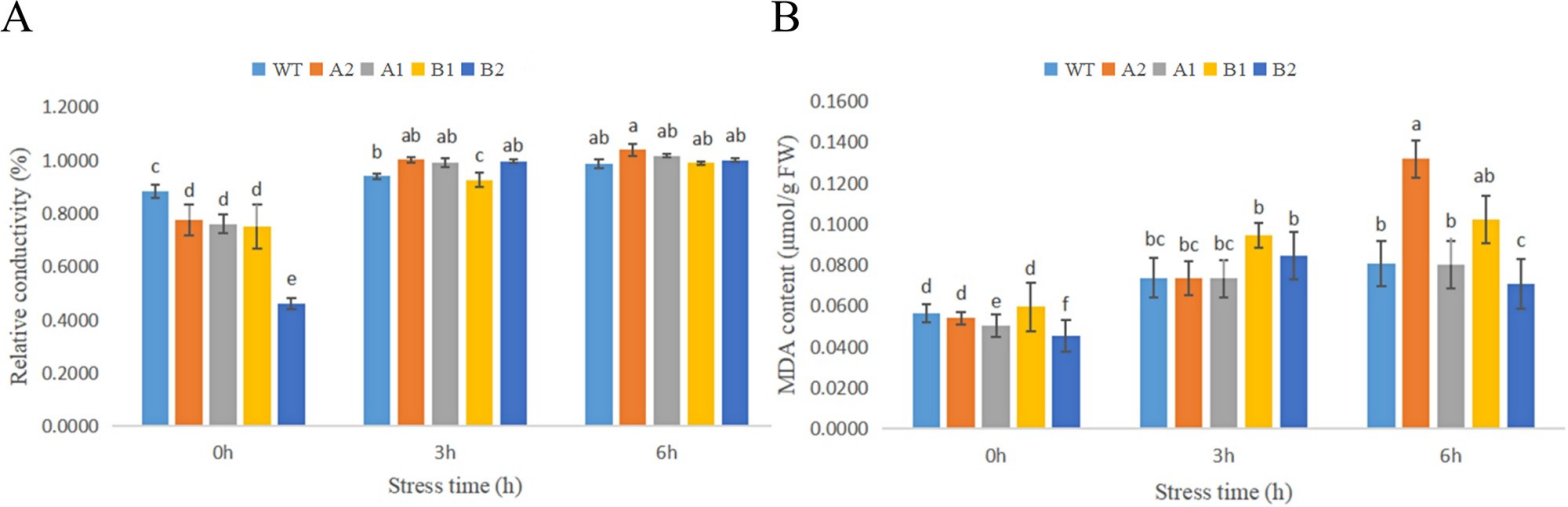
(A) Changes in the relative conductivity of chrysanthemum leaf cells under high-temperature treatment; (B) Changes in the MDA content in chrysanthemum under high-temperature treatment.

The relative electrolyte permeability of CmHSP17.9–1 and CmHSP17.9–2 transgenic chrysanthemum under heat stress was measured. The results are shown in Fig 14A. Under HT treatment, the relative conductivity of the chrysanthemum seedling leaves increased with the extension of heat stress time, indicating that the leaves were damaged by high temperature and that the cell permeability had increased. The relative electrolyte permeability of wild-type and transgenic plants increased, and there was no significant difference.

In the range of 0–6 h under 45°C stress, the change trend of the MDA content in the leaves of transgenic chrysanthemum seedlings was generally consistent with that of the control and increased with the extension of stress time. There was no significant difference in MDA content between wild-type and CmHSP17.9–1 transgenic plants under high-temperature stress for 3 h, and the MDA content of the B1 and B2 strains of CmHSP17.9–2 was higher than that of the control under high-temperature stress for 3 h. The MDA content of the A2 strain of CmHSP17.9–1 increased rapidly after 6 h of high-temperature stress. MDA is one of the products of membrane lipid peroxidation. The increase in MDA content indicated that the peroxidation of membrane lipids was enhanced and that the damage caused by high temperature to the cell structure was increased [30] (Fig 14B).

Overall, there was no significant difference between the damage caused by heat stress to the cell membrane system of CmHSP17.9–1 and CmHSP17.9–2 transgenic chrysanthemum and the control.

#### 3.6.4 Analysis of reactive oxygen accumulation in chrysanthemum under high-temperature stress

To clarify the high-temperature tolerance of the overexpression strains, the accumulation of two main reactive oxygen species, H_2_O_2_ and ${\text{O}}_{2}^{-}$, was detected by using diaminobenzidine (DAB) and nitroblue tetrazolium (NBT) staining.

**Fig 15:**
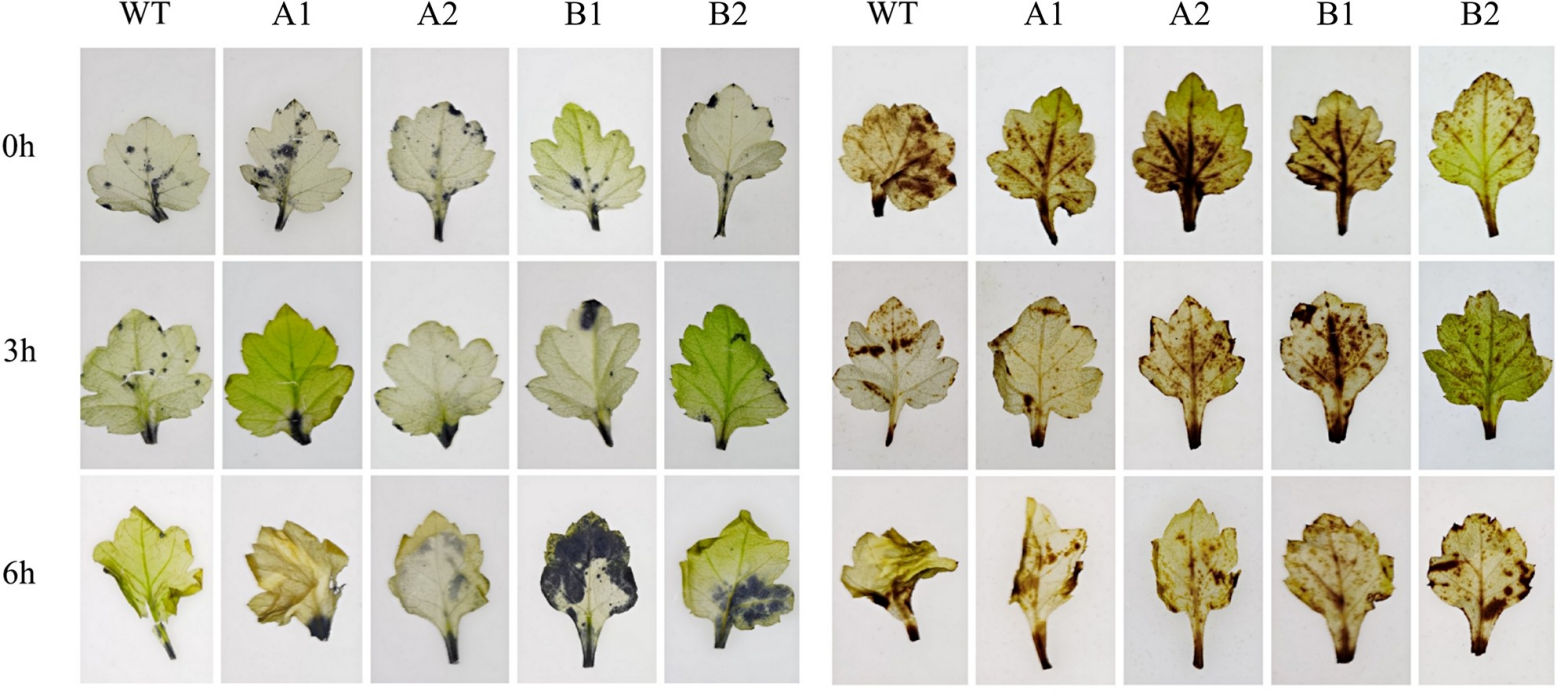
Accumulation of H_2_O_2_ and ${\text{O}}_{2}^{-}$ in chrysanthemum under high-temperature treatment. (NBT staining on the left and DAB staining on the right). This figure © 2024 by Qin Ling et al. is an open access article distributed under the terms of the Creative Commons Attribution License, which permits unrestricted use, distribution, and reproduction in any medium, provided the original author and source are credited.

It can be seen from the Fig 15 that with the extension of the stress time, the number of blue spots on the leaves of all strains began to increase, and the colour began to deepen gradually, indicating that there was reactive oxygen accumulation in these parts of the leaves. At 0 h, all strains showed no excessive accumulation of ${\text{O}}_{2}^{-}$. The blue colour of the leaves deepened after 3 hours of high-temperature stress, especially for the two strains with the CmHSP17.9–1 gene, which accumulated less ${\text{O}}_{2}^{-}$ than the control, and the difference between the two strains with the CmHSP17.9–2 gene and the control was not significant. After 6 h, the WT and CmHSP17.9–1 transgenic strains still did not exhibit excessive accumulation of ${\text{O}}_{2}^{-}$, while the two strains with the CmHSP17.9–2 gene showed multiple blue spots, indicating that ${\text{O}}_{2}^{-}$ accumulated a greater levels and that the plants suffered more damage from high temperature.

The DAB staining results showed many brown spots on the leaves of chrysanthemum, indicating the accumulation of H2O2 in these parts of the leaves. At 0 h, there were brown spots on the leaves to a similar degree, which may have been caused by some mechanical damage. After 3 hours of high-temperature stress, the brown spots of the transgenic strains were fewer than those of the control, except for the B1 strain with the CmHSP17.9–2 gene. After 6 hours, the spots of each strain increased, and their colour deepened; there was no significant difference between strains.

In conclusion, overexpression of CmHSP17.9–1 can reduce the accumulation of reactive oxygen species in chrysanthemum leaf cells at the early stage of high-temperature stress, thus reducing the damage caused by peroxidation to chrysanthemum. After overexpression of CmHSP17.9–2, the reduction in the accumulation of reactive oxygen was not obvious, especially the accumulation of ${\text{O}}_{2}^{-}$, which was consistent with the subsequent results for ascorbic acid peroxidase activity.

#### 3.6.5 Changes in antioxidant enzyme activity of chrysanthemum under high- temperature stress

**Fig 16:**
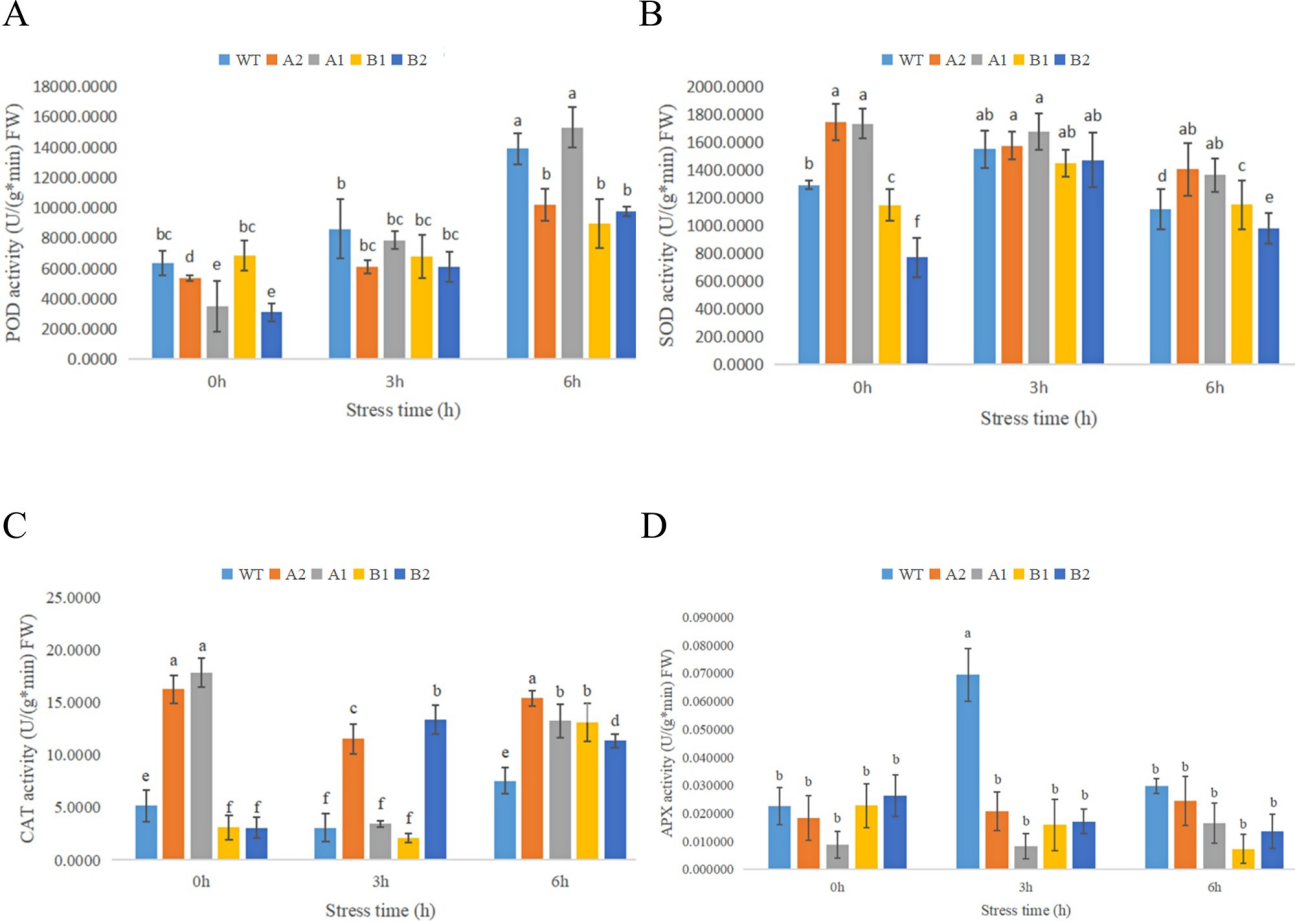
(A) Changes in the POD activity in chrysanthemum leaves under high-temperature treatment; (B) Changes in the SOD activity in chrysanthemum leaves under high-temperature treatment; (C) Changes in the CAT activity in chrysanthemum leaves under high-temperature treatment; (D) Changes in the APX activity in chrysanthemum leaves under high-temperature treatment.

As shown in the Fig 16, with increasing heat stress duration, the POD activity of the five chrysanthemum strains showed an upwards trend, indicating that at the early stage of heat stress, chrysanthemum seedlings could improve their POD enzyme activity to adapt to environmental stress through their own regulatory mechanism [31]. Among them, the POD activity of the A1 transgenic strain of CmHSP17.9–1 was significantly higher than that of the control after 6 hours of high-temperature stress. The POD activity of the B2 transgenic strain of CmHSP17.9–2 increased more to 1.44 times that of the control. Based on the results of other studies [16], it is speculated that POD activity changed slowly, and the other strains showed no significant difference or lower activity than the control due to insufficient stress duration [32]. Under high-temperature treatment, the activity of SOD in the leaves of chrysanthemum seedlings first increased and then decreased. Compared with the control, the difference in SOD activity of transgenic strains was not significant at 3 h under high-temperature stress, but it was significantly higher than the control after 6 h. After 3 hours of heat stress, the SOD activity of the leaves of the control decreased sharply, while the SOD activity of the leaves of the chrysanthemum seedlings overexpressing the genes decreased slightly. In general, the SOD activity of transgenic chrysanthemum was significantly higher than that of WT under high-temperature stress.

The activity of CAT decreased first and then increased. Compared with the control, the CAT activity of the transgenic strains increased more obviously. After 6 hours of high-temperature stress, the CAT activity of the transgenic strains was significantly higher than that of the control, i.e., 2.04, 1.75, 1.73 and 1.50 times higher than that of the control. The above results showed that the overexpression of the CmHSP17.9–1 and CmHSP17.9–2 genes in chrysanthemum could increase the activity of antioxidant enzymes to resist the oxidative damage caused by high-temperature stress.

Compared with the control, the APX activity of chrysanthemum seedling leaves decreased significantly after 3 hours of high-temperature treatment, and the difference was not significant after 6 hours. When the activity of ascorbic acid peroxidase (APX) is significantly increased, the production rate of superoxide anion radical (${\text{O}}_{2}^{-}$) will be significantly reduced, and the effect of lipid peroxidation will be weakened [33, 34]. The APX activity of the transgenic strains was lower than that of the wild type, which indicates that the production rate of superoxide anion free radicals (${\text{O}}_{2}^{-}$) increased, especially in the two strains with the CmHSP17.9–2 gene, which is consistent with the NBT staining results.

#### 3.6.6 Changes in osmoregulation substance content in chrysanthemum under high-temperature stress

**Fig 17:**
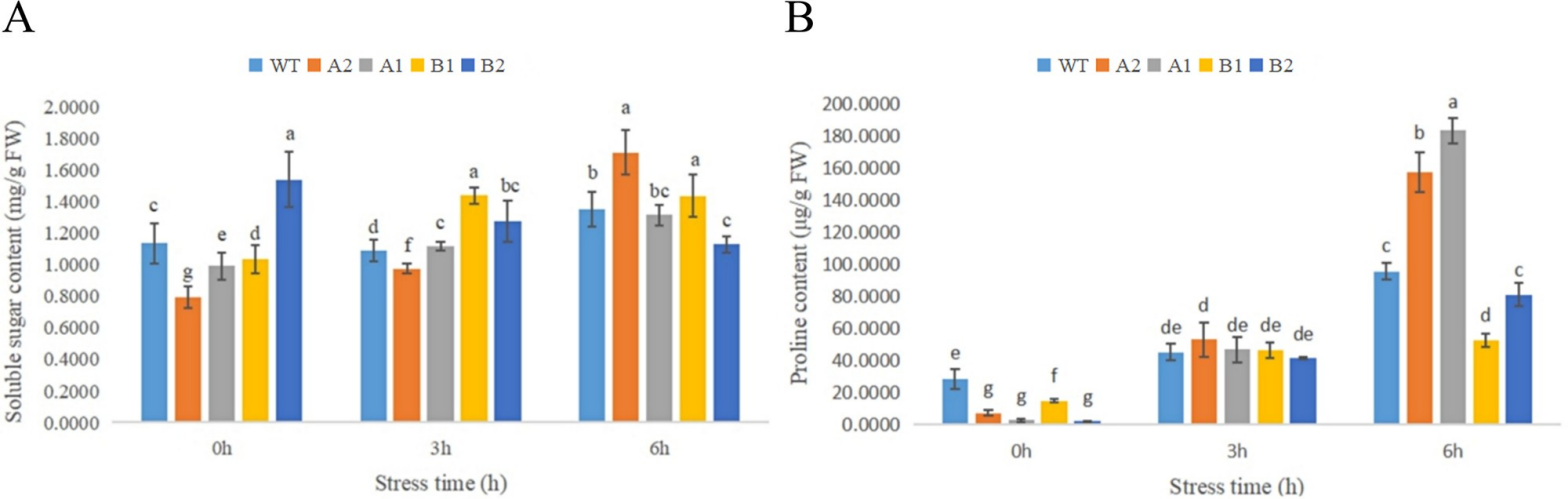
(A) Changes in the soluble sugar content in chrysanthemum leaves under high-temperature treatment; (B) Changes in the free proline content in chrysanthemum leaves under high-temperature treatment.

As shown in the Fig 17, under HT treatment, the soluble sugar content in the leaves of chrysanthemum seedlings increased with the extension of stress time, indicating that the leaves may resist high-temperature injury by accumulating soluble sugar [35]. Compared with the control, the soluble sugar content of the transgenic strains increased more significantly. The soluble sugar content of the A2 strain of CmHSP17.9–1 and the B1 strain of CmHSP17.9–2 was significantly higher than that of the control after 6 hours of high-temperature stress.

Compared with the control, the content of free proline in the leaves of chrysanthemum seedlings increased with the extension of high-temperature stress duration under HT treatment. The PRO content of the two transgenic strains with CmHSP17.9–1 was significantly higher than that of the control under high-temperature stress for 6 h, indicating that the antioxidant capacity of the transgenic plants under high-temperature stress was stronger than that of the control. The PRO content of CmHSP17.9–2 transgenic strains was not significantly different from that of the control at 3 h under high-temperature stress but was significantly lower than that of the control at 6 h.

## Discussion

### 4.1 Classification characteristics of the CmHSP17.9–1 and CmHSP17.9–2 proteins

According to the subcellular localization of proteins and the homology of amino acid sequences, sHSPs in plants can be divided into five major subfamilies: The CI, CII, CIII, CIV, CV, CVI and CVII subfamilies located in cytoplasm or nucleus; MI and MII subfamilies located in mitochondria; P subfamily located in plastids; ER subfamily located in the endoplasmic reticulum; Po subfamily located in peroxisomes [25]. CI sHSPs usually constitute the largest sHSP gene family in higher plants, while the CII gene family is usually small [36, 37].

The analysis of the conserved domains in the NCBI protein database showed that the 52–141 amino acid region of CmHSP17.9–1 and CmHSP17.9–2 was the typical ACD (alpha crystal domain) domain of HSP20 subfamily small molecular heat shock proteins. The results of the combined amino acid multiple sequence alignment showed that CmHSP17.9–1 and CmHSP17.9–2 belonged to the CII small molecular heat shock protein family. Tripp Eq [38] reported that the expression of CI sHSP rather than CII sHSP could enhance the protection of firefly luciferase introduced during heat stress in tomato (Solanum lycopersicum) protoplasts, indicating that CII sHSP may have a completely different function from molecular chaperone activity.

### 4.2 Small molecular heat shock proteins CmHSP17.9–1 and CmHSP17.9–2 are located in the cytoplas

The results of subcellular localization analysis showed that the CmHSP17.9-GFP fusion protein was located in the cytoplasm and was mostly aggregated in granules, especially around the nucleus, forming large aggregates that were not distributed in the nucleus. The analysis of the protein sequences of CmHSP17.9–1 and CmHSP17.9–2 showed that CmHSP17.9–1 and CmHSP17.9–2 did not have transmembrane structures or signal peptides and could not penetrate the cell membrane. This result is consistent with the previous localization results for OsHSP20 and NbHSP20 in rice, and this experiment showed that the granular structure formed by OsHSP20 and NbHSP20 in Benji tobacco could move and that the previously formed granular fluorescent aggregates may also disappear with the change [39]. Martin Haslbeck and others [40] also found that oligomeric sHSPs exist as dynamic sets, which may be crucial to their partner function.

### 4.3 Expression mode of CmHSP17.9–1 and CmHSP17.9–2 in chrysanthemum

Under high-temperature stress, various heat shock proteins and heat shock factors are activated or closed, forming a complex regulatory system to resist heat stress [41, 42]. CmHSP17.9–1 and CmHSP17.9–2 belong to the small molecular heat shock protein family (sHSPs). Many studies have confirmed that small molecular heat shock proteins (sHSPs), as molecular chaperones, play an important role in plant resistance to high-temperature heat injury, low-temperature cold injury and seed development, among which high-temperature stress is the most important factor. sHSPs cannot be detected under normal growth conditions, but stress conditions can activate their expression. Some studies have shown that under normal conditions, the content of sHSPs is very low, but under high-temperature stress, the content increases sharply in a short time, and more types of sHSPs are detected than other heat shock proteins [43].

The tissue-specific expression analysis of the genes showed that the expression levels of CmHSP17.9–1 and CmHSP17.9–2 in different organs were very different. The relative expression of CmHSP17.9–1 and CmHSP17.9–2 was the highest in the terminal buds, followed by the leaves, and the lowest in the stem. It is speculated that these genes are more likely to participate in heat tolerance reactions in terminal buds and leaves. The results of qRT-PCR analysis showed that both CmHSP17.9–1 and CmHSP17.9–2 could rapidly respond to high-temperature stress, and their expression was upregulated within 1 h. The upregulation amplitude in transgenic chrysanthemum was significantly greater than that in WT, indicating that heat shock rapidly induced the expression of CmHSP17.9–1 and CmHSP17.9–2. This is consistent with the results for expression of FaHSP17.8 and FaHSP17.9 induced by high temperature in tall fescue [44].

### 4.4 The heat tolerance of chrysanthemum overexpressing the CmHSP17.9–1 and CmHSP17.9–2 genes was enhanced

Phenotypic observation and physiological index determination after high-temperature stress showed that the curling and drooping of leaves of wild type chrysanthemum were more serious than those of transgenic chrysanthemum, indicating that the transgenic plants were less strongly affected by high temperature. Overexpression of the CmHSP17.9–1 and CmHSP17.9–2 genes in chrysanthemum could improve the high-temperature tolerance of chrysanthemum. Biosynthesis in plants depends on the enzymatic reaction of chlorophyll, which changes due to temperature differences [45]. Generally, excessively high or low temperatures affect the synthesis of chlorophyll. The optimum temperature for the enzymatic reaction of chlorophyll is 20–30°C, and the lower limit and upper limit of the temperature are 2–4°C and 40°C, respectively. Beyond this temperature range, chlorophyll synthesis is blocked [46]. In this study, the chlorophyll content of the four transgenic strains increased significantly compared with that of the control, indicating that under high-temperature stress, the chlorophyll loss in transgenic chrysanthemum was less than that in wild type chrysanthemum, and the ability to maintain chlorophyll was stronger. Under high-temperature stress, the activities of POD, SOD and CAT in transgenic chrysanthemum were significantly higher than those in WT, indicating that overexpression of the CmHSP17.9–1 and CmHSP17.9–2 genes in chrysanthemum could increase the activity of antioxidant enzymes to resist the oxidative damage caused by high-temperature stress. Measurement of the levels of soluble sugar and proline showed that the levels of osmoregulatory substances in the two CmHSP17.9–1 transgenic strains were significantly higher than that of the control under high-temperature stress, indicating that the antioxidant capacity of transgenic plants under high-temperature stress was stronger than that of the control. The difference between CmHSP17.9–2 transgenic strains and the control was relatively insignificant, which was weaker than the performance of CmHSP17.9–1 transgenic strains. The content of MDA and the relative permeability of the cell membrane of the overexpression and wild-type chrysanthemum strains did not show significant differences within 6 hours of high-temperature treatment, while the accumulation of the reactive oxygen species H_2_O_2_ and ${\text{O}}_{2}^{-}$ and the change in ascorbic acid peroxidase (APX) activity indicated that overexpression of CmHSP17.9–1 alleviated the accumulation of reactive oxygen species in the leaves of chrysanthemum at the early stage of high-temperature stress, thus reducing the damage caused by peroxidation. However, after overexpression of CmHSP17.9–2, the effect of reducing the accumulation of reactive oxygen species was not obvious, especially the accumulation of ${\text{O}}_{2}^{-}$ Some scholars have found that HSP family genes may make the overexpression strains more sensitive to high temperature through the influence of the reactive oxygen species pathway. It is speculated that this may be the cause of the accumulation of reactive oxygen species and peroxidation damage in the CmHSP17.9–2 overexpressing strains.

In summary, chrysanthemum plants overexpressing the CmHSP17.9–1 and CmHSP17.9–2 genes exhibited stronger tolerance than the wild type chrysanthemum after high-temperature treatment, and overexpression of CmHSP17.9–1 gene led to stronger heat tolerance than that of the CmHSP17.9–2 gene. The specific mechanism via which CmHSP17.9–1 and CmHSP17.9–2 respond to high temperature to regulate the tolerance of chrysanthemum is still unclear, and we will continue to explore the regulatory mechanism of chrysanthemum small molecular heat shock proteins through a series of biochemical molecular experiments.

## Conclusion

In this study, two small heat shock protein genes were isolated and named CmHSP17.9–1 and CmHSP17.9–2. The expression of CmHSP17.9–1 and CmHSP17.9–2 was found to have significant changes in response to high temperature by high-temperature treatment on the obtained overexpression strains. The analysis of physiological indicators showed that overexpression of CmHSP17.9–1 and CmHSP17.9–2 could improve the antioxidant and osmoregulation ability of transgenic plants, and thus improve the high-temperature tolerance of chrysanthemum plants. But the specific mechanism of how CmHSP17.9–1 and CmHSP17.9–2 respond to high temperature to regulate the tolerance of chrysanthemum is still unclear. These results have reference significance for the research on the heat tolerance of chrysanthemums at the protein and molecular levels, which will help elucidate the mechanism of high-temperature stress in chrysanthemums and provide theoretical basis for the introduction, cultivation, heat-tolerance breeding, and garden application of chrysanthemums. It provides a reference for the study of small molecular heat shock proteins in chrysanthemum, which were previously less studied.

## Supporting information

S1 Table Primers for gene amplification. (XLSX)

S2 Table Primers for the identification of positive strains. (XLSX)

S3 Table Primers for qRT-PCR reaction. (XLSX)

S4 Table GFP fusion protein expression vector primer. (XLSX)

S1 Raw images (PDF)
