# Supplementary figures and images for: Cloning and functional verification of the CmHSP17.9 gene from chrysanthemum
Source: PLoS One. 2024 May 8;19(5):e0301721. doi: 10.1371/journal.pone.0301721 (PMC11078346; doi:10.1371/journal.pone.0301721)

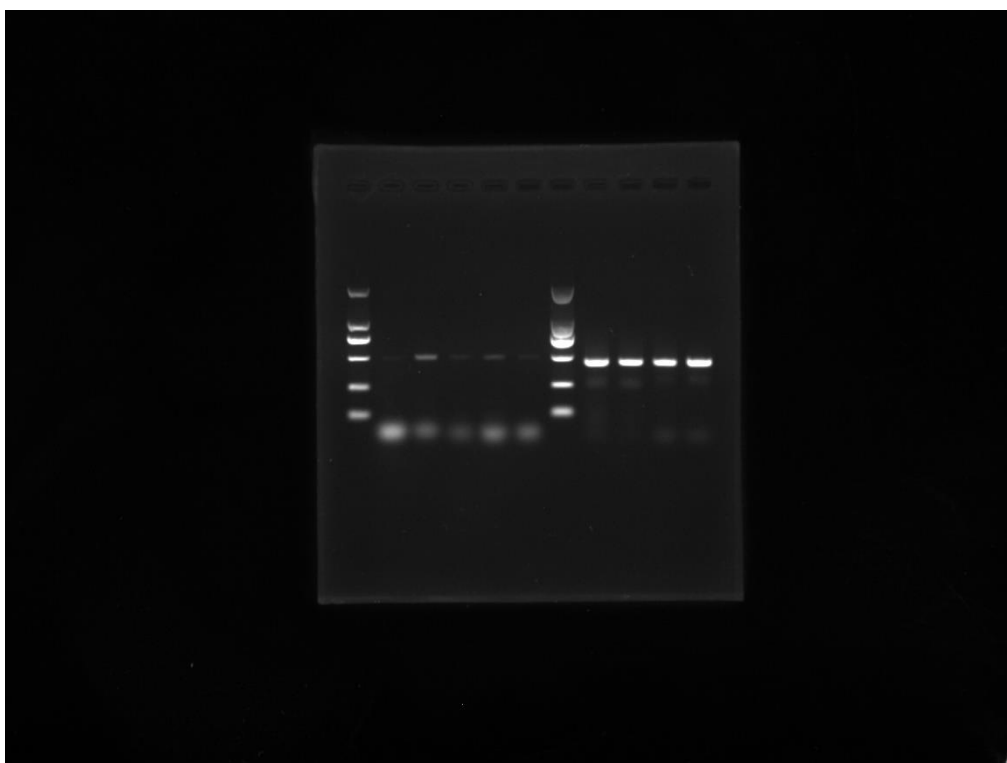

Original figure 1

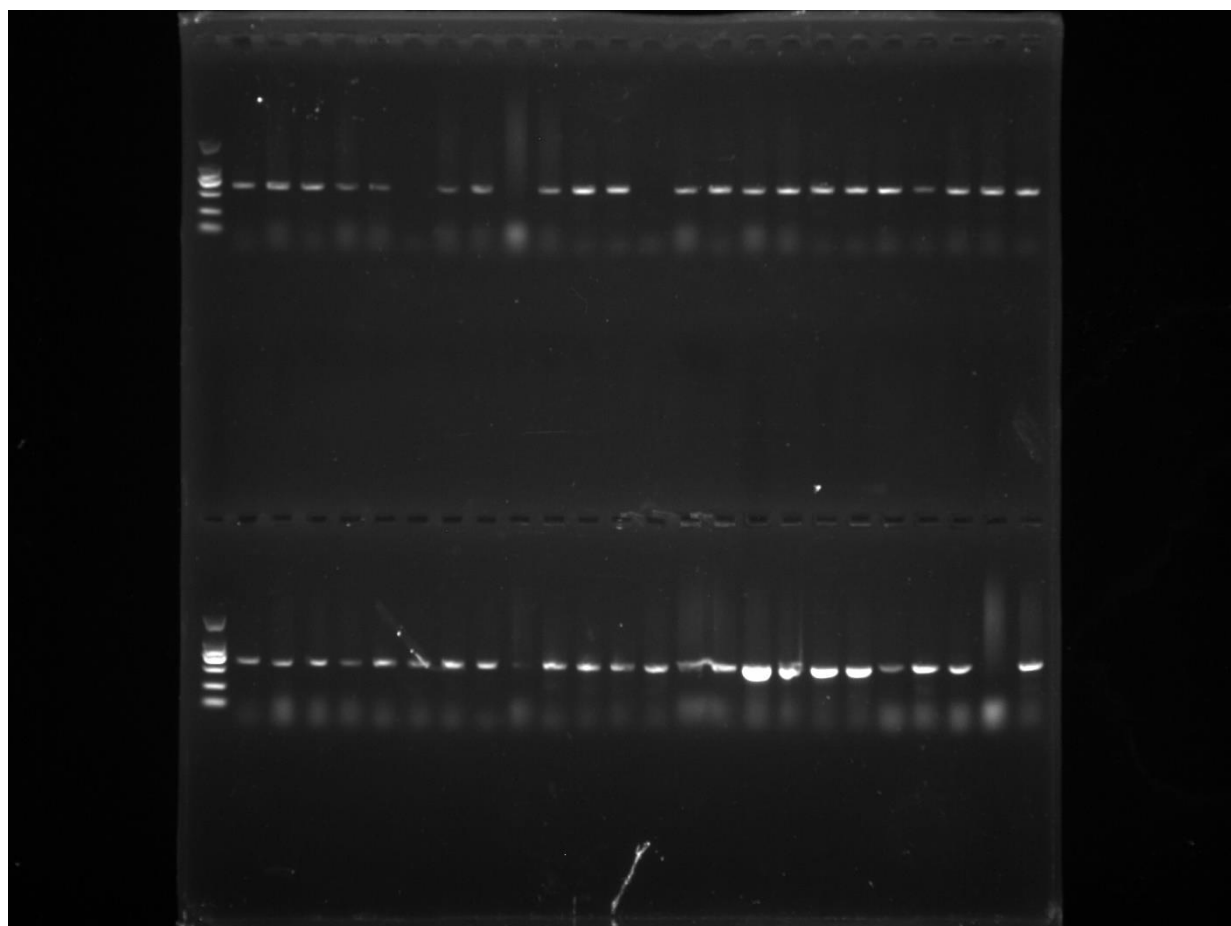

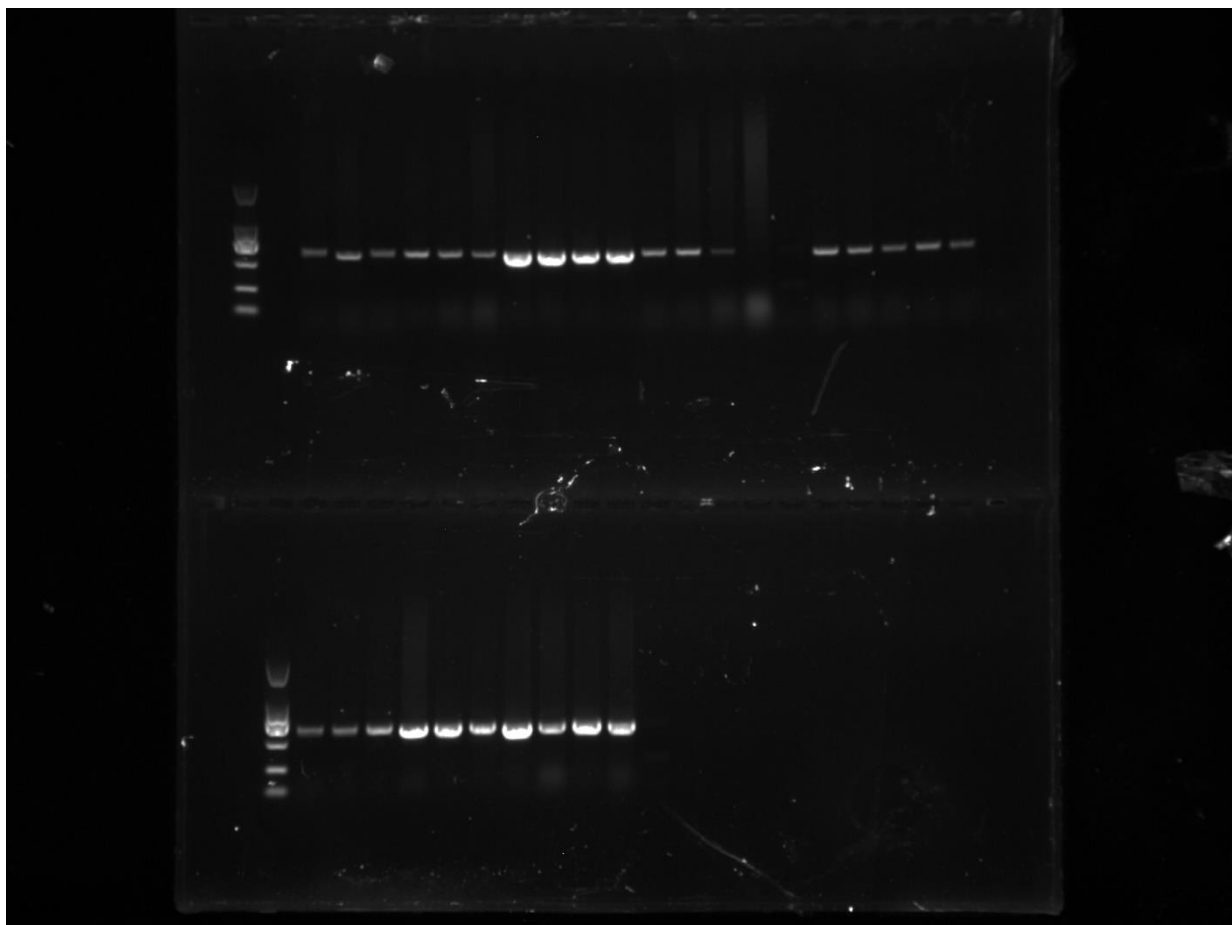

Original Figure 12

Supplement: S1 Raw images — (PDF) [file pone.0301721.s005.pdf]
